# Age-Related Differences in Response to High-Fat Feeding on Adipose Tissue and Metabolic Profile in ZDSD Rats

**DOI:** 10.1155/2013/584547

**Published:** 2013-05-20

**Authors:** Jeremy E. Davis, James Cain, William J. Banz, Richard G. Peterson

**Affiliations:** ^1^Animal Science, Food and Nutrition, Southern Illinois University, Carbondale, IL 62901-4317, USA; ^2^PreClinOmics Inc., Indianapolis, IN 46268-1649, USA

## Abstract

The recruitment of new fat cells through adipogenesis may prevent the development of obesity-related comorbidities. However, adipogenic capacity is markedly reduced in mature adults. This study examined how initiation of high-fat feeding at different phases of adulthood modified adipose tissue (AT) morphology and obesity phenotype in obese and diabetic Zucker Diabetic Sprague Dawley (ZDSD) rats. For this, rodents were provided high-fat diet (HFD) beginning at 63, 84, or 112 d after parturition until termination (*n* = 6). At termination, ZDSD rats fed HFD beginning at 63 d after parturition (early adulthood) exhibited greater body fat and lower lean mass without significant changes to energy intake or body weight. Moreover, early high fat feeding increased adipocyte size and number, whereas these effects were absent at 84 or 112 d after parturition. At 126 d after parturition, there were no detectable transcript differences in PPAR**γ** or C/EBP**α**. However, rodents provided HFD in early adolescence exhibited lower expression of canonical Wnt signaling intermediates. Corresponding with these changes was a marked reduction in AT-specific inflammation, as well as overall improvement in systemic glucose, lipid, and inflammatory homeostasis. Taken together, these data indicate that dietary regulation of adipocyte recruitment in adolescence may represent a major determinant of obesity phenotype.

## 1. Introduction

Obesity is commonly associated with the development of type 2 diabetes mellitus (T2DM) [1]. However, the occurrence of diabetic complications in obese subjects is highly variable. In fact, epidemiological data have indicated that ~30% of the obese population is classified as *metabolically healthy but obese* (MHO) [2, 3], which is characterized by normal insulin sensitivity and inflammatory profile regardless of excess body fat [4, 5]. Alternatively, there is a subgroup of normal weight subjects that exhibit metabolic dysfunction without increased adiposity [6, 7]. As such, the underlying factors involved in the development of obesity-related disease are poorly understood. 

Fat cell recruitment is a coordinately regulated process that involves activation of proadipogenic transcription factors, including peroxisome proliferator-activated receptor-*γ* (PPAR*γ*) and CCAAT-enhancer-binding protein-*α* (C/EBP*α*) [8, 9], as well as inhibition of antiadipogenic signals, including the canonical Wnt/*β*-catenin pathway [8, 10]. Thiazolidinediones (TZDs) represent a class of antidiabetic drugs that function through regulation of these pro- and antiadipogenic pathways [11, 12]. Similarly, high fat feeding is reported to enhance fat cell recruitment through activation and repression of PPAR*γ* and Wnt signaling, respectively [13, 14]. However, the ability to effectively induce adipogenesis with these mechanisms is markedly diminished with aging [15, 16]. This finding is supported by a stabilization of total fat cell number in older adults, irrespective of fluctuations with body weight [17]. In contrast, energy surplus during early maturation and young adulthood is commonly associated with morphological adaptations to both adipocyte size and number [18, 19].

The link between AT morphology and metabolic dysfunction has been investigated using a variety of preclinical models [20–22]. In particular, the Obese Zucker (OZR) and Zucker Diabetic Fatty (ZDF) rats are commonly used as a monogenic rodent model of obesity-related disease [23]. These rodents exhibit severe hyperphagia due to recessive homozygous mutation in the leptin receptor (*fa*). However, this mutation also represents a major limitation of these models as defects with leptin signaling are shown to directly impair adipogenic programming [24]. Consequently, morphological changes in AT of these rodents may not accurately reflect the pathophysiology of human obesity.

In light of such issues, the Zucker Diabetic Sprague Dawley/PreClinOmics (ZDSD/Pco) rat was developed. The ZDSD rat was generated by cross-breeding diet-induced obese (DIO) rats derived from the Charles River Laboratory: CD (Sprague Dawley-derived) strain with lean ZDF^*fa*/−^ rats. Selective inbreeding produced animals with a predisposition to DIO and potential to develop overt diabetes between 15 and 21 weeks of age with nutritional intervention [25]. In addition, these rodents display intact leptin signaling, as well as a more modest accumulation of body fat—approximately three times lower compared to OZR and ZDF rats. Therefore, our objective of this study was to use the ZDSD rat to determine the age-related effects of high-fat feeding on AT morphology on metabolic profile. 

## 2. Methods and Procedures

### 2.1. Animals, Diets, and Experimental Design

All experiments were conducted on male ZDSD/Pco rats (*N* = 24; PreClinOmics, Indianapolis, IN, USA). Animals were given unlimited access to standard low-fat diet ((LFD) Purina 5008; 16.7% kcal fat) postweaning (i.e., 21–28 d after parturition) and then provided high fat diet ((HFD) Research Diets, Inc. D12468; 47.7% kcal fat) beginning at either 63 d, 84 d, or 112 d until termination (i.e., 126 d after parturition). Additionally, a group of ZDSD rats were maintained on LFD for the duration of study. Early adulthood was classified as 63 d after parturition based on Hughes and Tanner [26]. All animals were housed under standard laboratory conditions with a 12 : 12 h light-dark cycle and a controlled room temperature (20-21°C). At 126 d after parturition, rats were fasted overnight and euthanized by CO_2_ asphyxiation. Serum and tissues were then collected, snap frozen in liquid nitrogen, and stored at −80°C for subsequent analysis. AT from the subcutaneous depot was collected from the dorsal scapular region. The protocol and all procedures were approved by the Institutional Animal Care and Use Committee of PreClinOmics and Southern Illinois University, Carbondale, IL, USA.

### 2.2. Anthropometric and Biochemical Measurements

Body composition (EchoMRI-700 Bioanalyzer, Echo Medical Systems, LLC) and blood glucose (Glucometer Elite, Mishawaka, IN, USA) were measured weekly. At termination, total serum cholesterol and triacylglyceride (TAG) were analyzed on a Beckman CX4 clinical analyzer with standard Beckman chemistries. Nonesterified fatty acids (NEFAs) were measured using an NEFA kit (Wako Chemicals, Richmond, VA, USA). Serum C-reactive protein (CRP), serum amyloid P (SAP), and IL-8 were all determined with appropriate assays (ALPCO Diagnostics, Salem, NH, USA). 

### 2.3. NF*κ*B p65 DNA Binding Activity

Nuclear protein was extracted from epididymal and subcutaneous AT (Nuclear Extraction Kit, Cayman Chemical, Ann Arbor, MI, USA). NF*κ*B p65 DNA binding activity was then determined with an enzyme-linked immunosorbent assay (Cayman Chemical). Briefly, a specific double-stranded DNA sequence containing the NF*κ*B response element was immobilized onto the bottom of wells of a 96-well plate. NF*κ*B p65 sequestered from the nuclear extract was detected by addition of a specific primary antibody directed against NF*κ*B p65. A secondary antibody conjugated to horseradish peroxidase was added to provide a sensitive colorimetric readout at 450 nm. 

### 2.4. Gene Expression Analysis

Total RNA was extracted from epidydimal and subcutaneous AT using Trizol reagent (Invitrogen, Carlsbad, CA, USA) and RNeasy mini columns (QIAGEN Inc., Valencia, CA, USA) according to the manufacturer's instructions. Purified mRNA was reversely transcribed into cDNA using RT^2^ PCR Array First Strand Kit (SABiosciences, Frederick, MD, USA) and immediately assayed for transcript abundance with customized RT^2^ Profiler PCR Arrays (SABiosciences) using gene-specific primers (proprietary primers, sequence not disclosed). cDNA was diluted into RT^2^ SYBR Green Master Mix (SABiosciences), and quantitative real time PCR was performed using a MyiQ Real-Time PCR Detection System (Bio-Rad, Hercules, CA, USA). Real-time PCRs were performed as follows: melting for 10 min at 95* *°C, 40 cycles of two-step PCR including melting for 15 sec at 95* *°C, and annealing for 1 min at 60* *°C. All cycle threshold (Ct) values of >35.0 were considered noncycling and removed from analysis. The raw data were analyzed using the ΔΔCt method following the manufacturer's instructions [27]. Data were presented as fold change relative to LFD treatment group. 

### 2.5. Histological Analysis

Subcutaneous and epidydimal AT was paraffin embedded and stained with hematoxylin and eosin. Morphological analysis was performed using an Olympus BX40 microscope (Olympus America Inc., Center Valley, PA, USA) in conjunction with Image-Pro Plus 5.1 (Media Cybernetics Inc., Bethesda, MD, USA) and Photoshop Extended CS4 Medical Analysis package (Adobe Systems Inc., San Jose, CA, USA). For the quantitation of number and size of adipocytes, the sectional areas of epididymal and subcutaneous AT in the hematoxylin- and eosin-stained preparations were analyzed with Leica QWin Standard software (Leica Microsystems Imaging Solutions, Cambridge, UK) as previously described [20]. 

### 2.6. Statistical Analysis

Data were tested for normality and analyzed using the mixed-model analysis with Bonferroni adjustment (SAS Institute Inc., Cary, NC, USA). Values are means ± standard error of mean (SEM). Post hoc comparisons were made using the least significant means (LSMEANS) separation with the PDIFF procedure. Differences among LSMEANS were considered significant at *P* < 0.05, and trends are noted when *P* < 0.10. This standard analysis was performed for all experiments and aims, unless otherwise specified.

## 3. Results

### 3.1. Body Composition and AT Morphology

**Figure 1:**
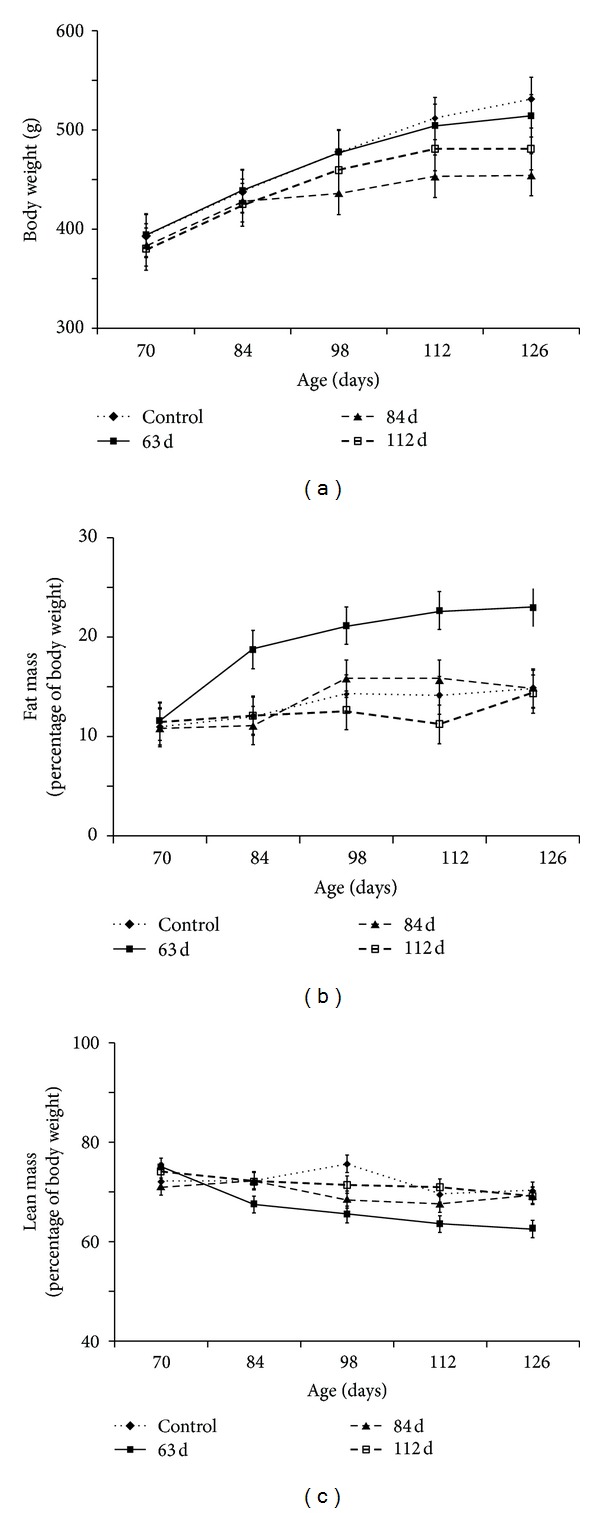
*Age-related effects of high-fat feeding on body weight and composition in male ZDSD rats*. Data were collected at two-week intervals from 70 d to 126 d after parturition. All graphed values represent means ± SE. (a) Body weight expressed as grams. (b) Total fat mass expressed as percent of body weight. (c) Total lean mass expressed as percent of body weight.

In this experiment, administration of HFD at 63 d after parturition (i.e., early adulthood) was associated with distinct changes in body composition without significant differences in overall body weight (Figure 1). At 126 d after parturition, adiposity was 8.2% greater, whereas lean mass was 6.7% lower with HFD at 63 d versus 84 or 112 d after parturition (Table 1, *P* < 0.05). Furthermore, we showed that changes in body composition were associated with modifications to AT morphology. Specifically, the mean adipocyte size and number were over 20% and 50% greater with HFD at 63 d after parturition versus all treatment groups (Table 1, *P* < 0.05). 

### 3.2. Adipogenic Programming

To identify potential signaling mechanisms associated with the observed morphological changes in AT, we examined mRNA abundance of several key adipogenic regulators in epidydimal and subcutaneous depots. We observed no marked difference in PPAR*γ* or C/EBP*α* transcript at 126 d after parturition (Table 2). Alternatively, transcript abundance of several genes associated with the canonical Wnt signaling network was significantly lower with HFD at 63 d after parturition (Table 2, *P* < 0.05). More specifically, the canonical Wnt ligands Wnt1, Wnt3a, and Wnt10b were all downregulated in subcutaneous and epidydimal AT with HFD at 63 d versus 112 d after parturition (*P* < 0.05). Expression of Wnt1 and Wnt3a was also lower in subcutaneous AT with initiation of HFD at 63 d versus 84 d after parturition (*P* < 0.05). Furthermore, transcript abundance of the canonical Wnt receptors Fzd1, Fzd2, and Fzd5 was lower in AT with initiation of high fat feeding at 63 d versus 112 d (*P* < 0.05). There were no significant differences in expression of the intracellular Wnt signaling intermediates GSK3*β*, *β*-catenin, or TCF/LEF in subcutaneous AT. Alternatively, transcript abundance of GSK3*β* and *β*-catenin was lower in epididymal AT with HFD at 63 d versus 112 d after parturition (*P* < 0.05).

### 3.3. AT Inflammation

**Figure 2:**
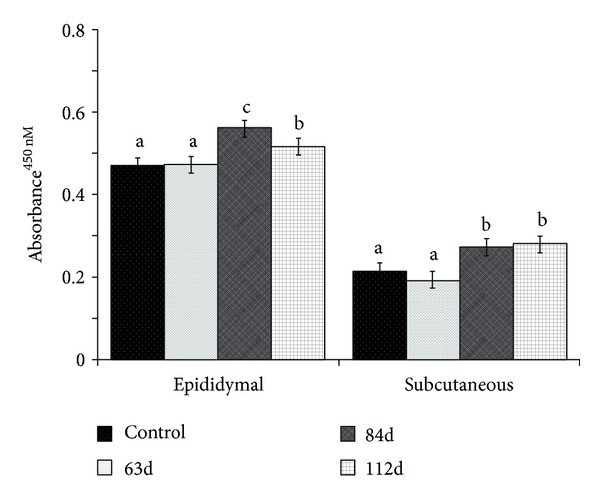
*Age-related effects of high-fat feeding on NF*κ*B p65 DNA binding activity in epididymal and subcutaneous AT of male ZDSD rats*. NF*κ*B p65 DNA binding activity was determined by an ELISA, according to the manufacturer's instructions (Cayman Chemical). Displayed values represent LSMEANS ± SE. Different letters represent significant differences between groups (*P* < 0.05).

AT dysfunction is associated with a proinflammatory state that contributes to an induction of canonical Wnt/*β*-catenin signaling [28]. Herein, NF*κ*B p65 DNA binding activity was 10–20% lower in epididymal AT and 40–45% lower in subcutaneous AT in rodents fed HFD beginning at 63 d versus 84 and 112 d after parturition (Figure 2, *P* < 0.05). The mRNA abundance of NF*κ*B was also moderately lower in epididymal AT with HFD at 63 d versus 112 d (Table 3, *P* < 0.05). Moreover, the expression of downstream targets of NF*κ*B was lower in AT with HFD at 63 d versus 112 d (Table 3). In particular, transcript abundance of the acute phase proteins CRP and SAP was lower with HFD at 63 d versus 112 d after parturition (*P* < 0.05). Similarly, MCP1 transcript abundance was markedly lower in subcutaneous and epididymal AT with HFD at 63 versus 112 d (*P* < 0.05). The mRNA abundance of the macrophage-specific marker F4/80 was also lower with HFD at 63 d versus 112 d after parturition (*P* < 0.05). 

### 3.4. Metabolic Profile

As discussed previously, not all obese individuals exhibit metabolic dysfunction [3–5, 29]. Consistent with a benign obesity phenotype, we showed that blood glucose concentration was 60–65% lower with HFD at 63 d versus 84 or 112 d after parturition (Table 4, *P* < 0.0001). Furthermore, several markers of dyslipidemia were lower with initiation of high-fat feeding in early adulthood (Table 4). In particular, serum cholesterol, NEFA, and TAG concentrations were 15%, 50%, and 60% lower with HFD at 63 d versus 84 or 112 d after parturition (*P* < 0.05). Moreover, the serum inflammatory markers SAP, CRP, and IL-8 were 7 to 20%, 21 to 60%, and 55 to 62% lower with high fat feeding at 63 d versus 84 or 112 d after parturition (Table 4, *P* < 0.05). 

## 4. Discussion 

In this study, we show that initiation of high fat feeding in early adulthood was associated with greater preservation of local and systemic glucose, lipid, and inflammatory homeostasis compared to ZDSD rats fed HFD beginning at 84 or 112 d after parturition. Although the specific mechanisms are not fully elucidated, high fat feeding in early adulthood increased both adipocyte size and number, which was associated with a marked reduction in antiadipogenic Wnt signaling intermediates. Collectively, our data indicate that exposure to HFD during early adulthood modifies AT morphology and protects against obesity-related disease.

The concept of a “metabolically healthy but obese” (MHO) phenotype has been recognized for several decades [30]. Although the specific mechanisms are unclear, it is now suggested that AT plasticity may be a primary determinant of obesity phenotype [31]. Consistent with this hypothesis, our data show that increased fat cell size and number were associated with maintenance of metabolic homeostasis in ZDSD rats. Kim et al. [32] similarly reported that fat cell hyperplasia was a central mitigator of metabolic dysfunction in obese transgenic mice. Additionally, synthetic PPAR*γ* agonists are reported to enhance glucose and lipid metabolism through recruitment of adipocytes or dedifferentiation of mature fat cells [33, 34]. We also cannot exclude the potential protective impact of a modest expansion in fat cell volume. For instance, enlargement of subcutaneous fat cells was associated with greater insulin sensitivity in patients treated with pioglitazone [35], and a greater number of small adipose cells in obese individuals have been associated with impaired insulin sensitivity and may indicate impaired adipogenesis [36]. It is therefore likely that both adipocyte hyperplasia and hypertrophy are important for preservation of metabolic homeostasis in obesity. 

Age-related impairment of fat cell recruitment is also implicated as a major determinant of obesity phenotype [37, 38]. Previous work [39–41] has suggested that adipocyte number is influenced primarily in infancy and childhood. More recently, Spalding and colleagues [17] used integration of 14 C (from nuclear bomb tests) in genomic DNA to demonstrate that adipocyte number becomes relatively stable in late adulthood, irrespective of fluctuations in fat mass. In this study, our data indicate that early adulthood may also be an important phase for regulation of fat cell recruitment. These data are also consistent with a previous study showing diet-induced adipocyte hyperplasia in rodents at approximately 55 d after parturition [42]. As such, early adulthood represents an important phase for diet-induced changes in AT morphology. 

The reduction in fat cell recruitment during late adulthood tightly corresponds with a decrease in expression or activation of PPAR*γ* [15, 43]. It is well recognized that PPAR*γ* acts as a master regulator for adipogenesis. As such, nearly all aspects of fat cell morphology are regulated by agonists and antagonists for this transcription factor [8, 9]. In our study, we observed no differences in PPAR*γ* mRNA abundance at 126 d after parturition. This may indicate that any changes in expression or activation occurred prior to termination. Consistent with this observation, previous studies have shown that induction of PPAR*γ* is markedly reduced in aged rodents [15, 16]. Although it is also possible that initiation of high-fat feeding prior to 63 d after parturition may have a more substantial impact on AT morphology, our data indicate regulation of PPAR*γ* in early adulthood is likely responsible for the increase in fat cell number.

Previous studies [44, 45] have shown that activation of canonical Wnt/*β*-catenin signaling augments adipocyte hypertrophy by antagonism of PPAR*γ* [44, 45]. Gustafson and Smith [34] suggested that activation of the Wnt signaling cascade exacerbated obesity-associated dysfunction through dedifferentiation of mature adipocytes. Consistent with these data, we showed that increased adipocyte number was associated with lower expression of the Wnt ligands, receptors, and signaling intermediates. Although the relationship between Wnt signaling and impaired adipogenesis has become evident, it remains unclear how this pathway may be differentially regulated in obesity. It was previously reported that increased inflammation inhibits adipocyte differentiation [46]. However, recent work suggested that increased TNF*α* and IL-6 enhanced fat cell hypertrophy through stimulation of the canonical Wnt/*β*-catenin pathway [28]. Herein, we similarly showed that reduced Wnt signaling was associated with lower NF*κ*B activation and proinflammatory gene expression in AT of obese ZDSD rats. Additional studies will need to determine whether dietary regulation of adipocyte recruitment in early adolescence is due to modification of Wnt/*β*-catenin signaling or inflammatory status. 

In summary, we have used a novel physiologically relevant preclinical model to determine how obesity phenotype is influenced by initiation of high-fat feeding at different phases of adulthood. Our findings suggest that administration of HFD in early adulthood maintained glucose, lipid, and inflammatory profiles through expansion of fat cell size and number. Although there were no changes in proadipogenic factors, we observed a marked reduction in antiadipogenic canonical Wnt/*β*-catenin signaling intermediates. Collectively, these data establish that dietary regulation of adipogenesis in early adulthood may help preserve metabolic homeostasis in obesity. 

## Figures and Tables

**Table 1:** Body composition and AT morphology in ZDSD rats fed HFD beginning at 63, 84, and 112 d after parturition.

	LFD	HFD 63 d	HFD 84 d	HFD 112 d
Body weight (g)	531 ± 27.4	514 ± 27.4	454 ± 27.4	480 ± 27.4
Fat mass^†^	14.8 ± 2.54^a^	23.0 ± 2.54^b^	14.8 ± 2.54^a^	14.3 ± 2.54^a^
Lean mass^†^	70.2 ± 1.79^a^	62.5 ± 1.79^b^	69.3 ± 1.79^a^	69.2 ± 1.79^a^
Water mass^†^	59.2 ± 2.35	55.3 ± 2.35	58.0 ± 2.35	57.9 ± 2.35
Subcutaneous adipocyte^‡^				
Area (*μ*m^2^)	8318 ± 807^ab^	10009 ± 931^b^	6360 ± 807^a^	6869 ± 807^a^
Number	371 ± 76.7^a^	647 ± 88.5^b^	288 ± 76.7^a^	266 ± 76.7^a^
Epididymaladipocyte^‡^				
Adipocyte area (*μ*m^2^)	9368 ± 1131^ab^	11356 ± 1131^b^	8530 ± 1131^a^	8520 ± 1131^a^
Number	149 ± 40.3^a^	270 ± 40.3^b^	137 ± 43.3^a^	128 ± 43.3^a^

*Letters represent significant differences among means as determined by post hoc comparison (*P* < 0.05).

^†^Data presented as percent of total body weight.

^‡^Determined by analysis of fixed area on H & E stained slides.

**Table 2:** Transcript abundance of Wnt signaling intermediates in AT depots of ZDSD rats fed HFD beginning at 63, 84, or 112 d after parturition.

	Subcutaneous				Epididymal	
	HFD 63d	HFD 84 d	HFD 112 d	HFD 63 d	HFD 84 d	HFD 112 d
Adipogenic transcription factors						

PPAR*γ*	1.18	1.56	1.23	1.17	1.20	1.43
C/EBP*α*	1.14	1.18	1.15	0.93	1.20	1.26

Wnt ligands						

Wnt1	0.33^a^	0.82^b^	0.88^b^	0.88^a^	1.56^a^	5.36^b^
Wnt3a	0.92^a^	1.95^b^	3.02^c^	1.12^a^	1.32^a^	3.92^b^
Wnt10b	0.62^a^	0.44^a^	2.97^b^	0.78^a^	0.47^a^	3.94^b^

Extracellular Wnt receptors						

Fzd1	0.93^a^	1.17^ab^	1.93^b^	1.29	1.45	1.44
Fzd2	0.53^a^	1.00^ab^	1.82^b^	2.69^a^	2.51^a^	10.74^b^
Fzd5	1.06^a^	1.46^ab^	1.95^b^	0.62^a^	1.08^ab^	1.38^b^

Intracellular Wnt signaling intermediates						

Gsk3*β*	1.02	1.43	0.89	4.39^a^	4.17^a^	8.20^b^
*β*-catenin	1.09	1.32	1.34	2.44^a^	2.41^a^	6.44^b^
TCF/LEF	0.62	0.82	0.78	0.85	1.28	1.27

*Data are expressed as fold change relative to animals fed LFD. Letters represent significant difference among treatment groups as determined by comparison of normalized Ct values (target Ct-housekeeping Ct) with paired *t*-test.

**Table 3:** Transcript abundance of proinflammatory factors in AT depots of ZDSD rats fed HFD beginning at 63, 84, or 112 d after parturition.

	Subcutaneous			Epididymal		
	HFD 63d	HFD 84 d	HFD 112 d	HFD 63 d	HFD 84 d	HFD 112 d
CRP	0.86^a^	1.40^ab^	3.22^b^	1.73^a^	1.80^a^	6.36^b^
SAP	0.97^a^	1.33^ab^	2.06^b^	2.25^a^	2.74^a^	7.50^b^
MCP1	0.83^a^	2.02^b^	2.45^b^	4.31^a^	4.36^a^	14.00^b^
NF*κ*B	0.78	1.24	1.36	2.24^a^	2.66^a^	5.96^b^
F4/80	0.91^a^	1.13^a^	2.46^b^	0.54^a^	1.40^ab^	1.95^b^

*Data are expressed as fold change relative to animals fed LFD. Letters represent significant difference among treatment groups as determined by comparison of normalized Ct values (target Ct-housekeeping Ct) with paired *t*-test.

**Table 4:** Systemic metabolic profile in ZDSD rats fed HFD beginning at 63, 84, or 112 d after parturition.

	LFD	HFD 63 d	HFD 84 d	HFD 112 d
Glucose (mmol/L)	10.33 ± 3.52^a^	8.94 ± 3.52^a^	24.27 ± 3.52^b^	26.02 ± 3.52^b^
TAG (mmol/L)	4.88 ± 7.50^a^	8.19 ± 7.50^a^	33.56 ± 7.50^b^	22.85 ± 7.50^b^
Cholesterol (mmol/L)	4.41 ± 0.10^a^	3.32 ± 0.10^b^	3.84 ± 0.10^c^	4.02 ± 0.10^c^
NEFA (mEq/L)	0.52 ± 0.06^a^	0.27 ± 0.06^b^	0.54 ± 0.06^c^	0.61 ± 0.06^c^
CRP (nmol/L)	16.22 ± 1.41^a^	4.39 ± 1.41^b^	12.85 ± 1.41^a^	3.60 ± 1.41^b^
SAP (*μ*mol/L)	2.79 ± 0.13^a^	1.58 ± 0.13^c^	2.00 ± 0.13^ab^	1.71 ± 0.13^bc^
IL-8 (pmol/L)	84.2 ± 17.5^a^	28.2 ± 17.5^b^	62.7 ± 17.5^ab^	74.9 ± 17.5^a^

*Letters represent significant differences among means as determined by post hoc comparison.

## References

[1] Smyth S, Heron A (2006). Diabetes and obesity: the twin epidemics. *Nature Medicine*.

[2] Shea JL, Randell EW, Sun G (2011). The prevalence of metabolically healthy obese subjects defined by BMI and dual-energy X-ray absorptiometry. *Obesity*.

[3] Wildman RP, Muntner P, Reynolds K (2008). The obese without cardiometabolic risk factor clustering and the normal weight with cardiometabolic risk factor clustering: prevalence and correlates of 2 phenotypes among the US population (NHANES 1999–2004). *Archives of Internal Medicine*.

[4] Stefan N, Kantartzis K, Machann J (2008). Identification and characterization of metabolically benign obesity in humans. *Archives of Internal Medicine*.

[5] Shin MJ, Hyun YJ, Kim OY, Kim JY, Jang Y, Lee JH (2006). Weight loss effect on inflammation and LDL oxidation in metabolically healthy but obese (MHO) individuals: low inflammation and LDL oxidation in MHO women. *International Journal of Obesity*.

[6] Kelishadi R, Cook SR, Motlagh ME (2008). Metabolically obese normal weight and phenotypically obese metabolically normal youths: The CASPIAN Study. *Journal of the American Dietetic Association*.

[7] Succurro E, Marini MA, Frontoni S (2008). Insulin secretion in metabolically obese, but normal weight, and in metabolically healthy but obese individuals. *Obesity*.

[8] Ross SE, Hemati N, Longo KA (2000). Inhibition of adipogenesis by Wnt signaling. *Science*.

[9] Kawai M, Mushiake S, Bessho K (2007). Wnt/Lrp/beta-catenin signaling suppresses adipogenesis by inhibiting mutual activation of PPARgamma and C/EBPalpha. *Biochemical and Biophysical Research Communications*.

[10] Bennett CN, Ross SE, Longo KA (2002). Regulation of Wnt signaling during adipogenesis. *The Journal of Biological Chemistry*.

[11] Gustafson B, Eliasson B, Smith U (2010). Thiazolidinediones increase the wingless-type MMTV integration site family (WNT) inhibitor Dickkopf-1 in adipocytes: a link with osteogenesis. *Diabetologia*.

[12] Luand D, Carson DA (2010). Repression of beta-catenin signaling by PPAR gamma ligands. *European Journal of Pharmacology*.

[13] Vidal-Puig A, Jimenez-Liñan M, Lowell BB (1996). Regulation of PPAR *γ* gene expression by nutrition and obesity in rodents. *Journal of Clinical Investigation*.

[14] Chen JR, Lazarenko OP, Wu X (2010). Obesity reduces bone density associated with activation of PPARgamma and suppression of Wnt/beta-catenin in rapidly growing male rats. *PLoS ONE*.

[15] Karagiannides I, Tchkonia T, Dobson DE (2001). Altered expression of C/EBP family members results in decreased adipogenesis with aging. *American Journal of Physiology*.

[16] Schipper BM, Marra KG, Zhang W, Donnenberg AD, Rubin JP (2008). Regional anatomic and age effects on cell function of human adipose-derived stem cells. *Annals of Plastic Surgery*.

[17] Spalding KL, Arner E, Westermark PO (2008). Dynamics of fat cell turnover in humans. *Nature*.

[18] Knittle JL, Timmers K, Ginsberg-Fellner F (1979). The growth of adipose tissue in children and adolescents. Cross-sectional and longitudinal studies of adipose cell number and size. *Journal of Clinical Investigation*.

[19] Prins JB, O’Rahilly S (1997). Regulation of adipose cell number in man. *Clinical Science*.

[20] Okuno A, Tamemoto H, Tobe K (1998). Troglitazone increases the number of small adipocytes without the change of white adipose tissue mass in obese Zucker rats. *Journal of Clinical Investigation*.

[21] Sugii S, Olson P, Sears DD (2009). PPARgamma activation in adipocytes is sufficient for systemic insulin sensitization. *Proceedings of the National Academy of Sciences of the United States of America*.

[22] Berk PD, Zhou SL, Kiang CL, Stump D, Bradbury M, Isola LM (1997). Uptake of long chain free fatty acids is selectively up-regulated in adipocytes of zucker rats with genetic obesity and non-insulin-dependent diabetes mellitus. *The Journal of Biological Chemistry*.

[23] Peterson R, Shafrir E (2008). The Zucker Diabetic fatty (ZDF) rat—lessons from a leptin receptor defect diabetic model. *Animal Models of Diabetes, Frontiers in Research*.

[24] Wagoner B, Hausman DB, Harris RBS (2006). Direct and indirect effects of leptin on preadipocyte proliferation and differentiation. *American Journal of Physiology*.

[25] Reinwald S, Peterson RG, Allen MR, Burr DB (2009). Skeletal changes associated with the onset of type 2 diabetes in the ZDF and ZDSD rodent models. *American Journal of Physiology*.

[26] Hughes PC, Tanner JM (1970). The assessment of skeletal maturity in the growing rat. *Journal of Anatomy*.

[27] Artaza JN, Bhasin S, Mallidis C, Taylor W, Ma K, Gonzalez-Cadavid NF (2002). Endogenous expression and localization of myostatin and its relation to myosin heavy chain distribution in C2C12 skeletal muscle cells. *Journal of Cellular Physiology*.

[28] Gustafson B, Smith U (2006). Cytokines promote Wnt signaling and inflammation and impair the normal differentiation and lipid accumulation in 3T3-L1 preadipocytes. *The Journal of Biological Chemistry*.

[29] Shea JL, Randell EW, Sun G (2011). The prevalence of metabolically healthy obese subjects defined by BMI and dual-energy X-ray absorptiometry. *Obesity*.

[30] Sims EA, Brodoff BN, Bleicher SJ (1982). Characterization of the syndromes of obesity. *Diabetes Mellitus and Obesity*.

[31] Denisand GV, Obin MS (2013). ‘Metabolically healthy obesity’: origins and implications. *Molecular Aspects of Medicine*.

[32] Kim JY, Van De Wall E, Laplante M (2007). Obesity-associated improvements in metabolic profile through expansion of adipose tissue. *Journal of Clinical Investigation*.

[33] De Souza CJ, Eckhardt M, Gagen K (2001). Effects of pioglitazone on adipose tissue remodeling within the setting of obesity and insulin resistance. *Diabetes*.

[34] Gustafson B, Smith U (2010). Activation of canonical wingless-type MMTV integration site family (Wnt) signaling in mature adipocytes increases *β*-catenin levels and leads to cell dedifferentiation and insulin resistance. *The Journal of Biological Chemistry*.

[35] Koenen TB, Tack CJ, Kroese JM (2009). Pioglitazone treatment enlarges subcutaneous adipocytes in insulin-resistant patients. *Journal of Clinical Endocrinology and Metabolism*.

[36] McLaughlin T, Sherman A, Tsao P (2007). Enhanced proportion of small adipose cells in insulin-resistant vs insulin-sensitive obese individuals implicates impaired adipogenesis. *Diabetologia*.

[37] Fabbrini E, Magkos F, Mohammed BS (2009). Intrahepatic fat, not visceral fat, is linked with metabolic complications of obesity. *Proceedings of the National Academy of Sciences of the United States of America*.

[38] Srdic B, Stokic E, Korac A (2010). Morphological characteristics of abdominal adipose tissue in normal-weight and obese women of different metabolic profiles. *Experimental and Clinical Endocrinology & Diabetes*.

[39] Ravelli GP, Stein ZA, Susser MW (1976). Obesity in young men after famine exposure in utero and early infancy. *The New England Journal of Medicine*.

[40] Rolland Cachera MF, Deheeger M, Bellisle F (1984). Adiposity rebound in children: a simple indicator for predicting obesity. *American Journal of Clinical Nutrition*.

[41] Brook CG, Lloyd JK, Wolf OH (1972). Relation between age of onset of obesity and size and number of adipose cells. *British Medical Journal*.

[42] Pouteau E, Turner S, Aprikian O (2008). Time course and dynamics of adipose tissue development in obese and lean Zucker rat pups. *International Journal of Obesity*.

[43] Hotta K, Bodkin NL, Gustafson TA, Yoshioka S, Ortmeyer HK, Hansen BC (1999). Age-related adipose tissue mRNA expression of ADD1/SREBP1, PPAR*γ*, lipoprotein lipase, and GLUT4 glucose transporter in rhesus monkeys. *Journals of Gerontology A*.

[44] Yang X, Jansson PA, Nagaev I (2004). Evidence of impaired adipogenesis in insulin resistance. *Biochemical and Biophysical Research Communications*.

[45] Isakson P, Hammarstedt A, Gustafson B, Smith U (2009). Impaired preadipocyte differentiation in human abdominal obesity: role of Wnt, tumor necrosis factor-*α*, and inflammation. *Diabetes*.

[46] Gustafson B, Gogg S, Hedjazifar S (2009). Inflammation and impaired adipogenesis in hypertrophic obesity in man. *American Journal of Physiology*.

